# Integrating multiple seismic attributes for fault detection using a new hybrid machine learning

**DOI:** 10.1038/s41598-025-26889-y

**Published:** 2025-11-28

**Authors:** Hadi Esmaeili, Majid Bagheri, Shamseddin Esmaeili

**Affiliations:** 1 Seismology, Institute of Geophysics, University of Tehran, Tehran, Iran; 2 Institute of Geophysics, University of Tehran, Tehran, Iran; 3 Razi University, Kermanshah, Iran

**Keywords:** Faults, New hybrid machine, Seismic attributes, MLP, SVM, Solid Earth sciences, Engineering

## Abstract

This paper proposes a novel hybrid approach for fault detection in seismic data by integrating multiple seismic features using a novel hybrid machine. This hybrid method introduces the integration of Multilayer Perceptron (MLP) neural networks with Support Vector Machines (SVM). The main objective of this study is to enhance the analysis and recognition of fault patterns in seismic data while reducing detection errors. The dataset consists of two-dimensional synthetic and real seismic data and their corresponding labels. Various seismic features, such as the gray-level co-occurrence matrix (GLCM), and features derived from ant tracking, chaos, variance, sweetness, correlation, slope direction, and energy, are extracted and normalized. These features are subsequently used to train MLP and SVM models for fault detection. SVM, a supervised learning method, operates by determining a hyperplane that maximizes the separation between data classes. In contrast, MLP, an artificial neural network, uses multiple layers to optimize weights and capture complex data relationships. The performance of each model is evaluated using experimental data, and its corresponding accuracies are calculated. Finally, the predictions from both models are combined to improve the overall fault detection accuracy. The results show that this combined approach significantly increases the fault detection accuracy compared to the independent application of each model.

**Supplementary Information:**

The online version contains supplementary material available at 10.1038/s41598-025-26889-y.

## Introduction

Oil fields development and production wells drilling remain critical strategies in oil-rich countries^1^. However, hydrocarbon exploration and the development of carbonate reservoirs are inherently complex due to inadequate seismic imaging and reservoir heterogeneity arising from diagenetic variations^2^. Consequently, identifying faulted zones is vital for determining optimal drilling locations^3^. Detecting faults and fractures is an essential task throughout all stages of oilfield operations, including exploration, extraction, and production. Traditional methods require interpreters to spend considerable time visually identifying faults and fractures, followed by manual interpretation. When data quality is poor or geological formations are structurally complex, such identification can become challenging or even infeasible^4^. Manual interpretation of faults and fractures is often fraught with high uncertainty, particularly in basins with limited seismic data^5^. For instance, when faults align parallel to the strike, their detection becomes more difficult due to the overlap of fault lineaments with bedding lineaments.

Accurate fault identification and localization in seismic data are among the most critical steps in geophysical analysis. Faults, recognized as zones of structural weakness, play a significant role in assessing earthquake-prone areas. Given the complexity and volume of seismic data, advanced analytical methods are indispensable. This study investigates the application of MLP and SVM techniques for fault detection in seismic datasets, utilizing a range of extracted seismic attributes to achieve greater precision.

The Multi-Layer Perceptron (MLP) neural network was first introduced by Frank Rosenblatt in the 1960s. Initially limited to solving linear problems, its capabilities expanded significantly in the 1980s with the advent of the backpropagation algorithm, developed by David Rumelhart and colleagues, enabling the training of multi-layered networks. Today, MLP stands as a cornerstone of artificial intelligence and machine learning. Soft computing techniques are increasingly employed for reservoir characterization, with notable contributions from researchers such as Tanner^7^, Roberts^12^, Tingdahl et al.^13^, Aminzadeh et al.^14^, Ashraf et al.^15^, and Xiao et al.^16^. Recent advancements in hardware and software have further accelerated the adoption and evolution of these methodologies.

These recent contributions highlight the dynamic evolution of seismic fault detection techniques, with deep learning methods providing powerful alternatives. Nevertheless, such models often demand large labeled datasets, high computational resources, and GPU infrastructure, making them challenging to implement in all research and industry contexts. Against this background, our proposed hybrid approach based on MLP and SVM offers a simpler and more resource-efficient solution for 2D seismic datasets, while still achieving competitive accuracy compared with recent deep learning frameworks.

## Methodology

This research employs a hybrid approach combining MLP and SVM algorithms, implemented using MATLAB software. Initially, a suite of seismic attributes, including instantaneous phase and frequency, GLCM matrices (contrast, correlation, energy, and homogeneity), ant-tracking, curvature, edge detection (Sobel and Canny), dip, and azimuth, is extracted from the seismic data and normalized. Following extensive testing, frequency, GLCM, and edge-detection features were selected as inputs for the algorithms in this study, though feature selection may vary depending on the dataset. The data is then divided into training and testing sets, with MLP and SVM models trained using the training data. SVM identifies the optimal hyperplane to separate data classes, while MLP learns intricate data relationships through multiple layers. Model performance is evaluated on the test set, and accuracy is computed. Below, we briefly discuss key seismic attributes that yielded superior results in this study.

### Curvature attribute

Curvature measures the degree of bending or angular change at boundaries, proving effective for identifying structures with sharp variations, such as faults. In seismic and geological analysis, curvature attributes aid in interpreting seismic reflections and detecting features like faults and folds. This is achieved by fitting a quadratic surface, expressed mathematically, to seismic data points. For a given point and its eight neighbors, the local curvature is estimated using Eq. (1).

### Gray-level co-occurrence matrix (GLCM)

GLCM is a statistical tool that analyzes the co-occurrence of pixel intensities in an image and extracts textural features critical for fault detection. Key features include contrast, correlation, energy, and homogeneity, Eqs. (2–4)^19^.

### Ant-tracking attribute

Inspired by ants’ collective behavior, the ant-tracking algorithm identifies prominent paths or boundaries in images, making it highly effective for detecting faults and fractures. Rather than relying on a specific mathematical formula, it operates based on defined movement rules, prioritizing regions with distinct edges, Eq. (5)^20,21^.

### Dip and azimuth attributes

**Dip**: This attribute measures the angle of inclination of a subsurface layer relative to the horizontal plane, calculated from changes in depth or reflection time across directions. It is instrumental in characterizing geological structures like faults, Eq. (6).

**Azimuth**: Representing the direction of maximum dip, azimuth indicates the orientation or extent of geological features derived from horizontal coordinate variations, Eq. (7)^22^.

### Chaos

The Chaos attribute measures the degree of structural disorder in seismic data. It is particularly useful for identifying fault zones, fractures, and chaotic depositional environments, Eq. (8)^4^.

### Variance

Variance measures the lateral changes in seismic amplitude, highlighting discontinuities such as faults and stratigraphic features, Eq. (9)^4^.

### Sweetness (sweet)

Sweetness is the ratio of instantaneous amplitude to the square root of instantaneous frequency. It is commonly used to identify hydrocarbon reservoirs, Eq. (10)^23^.

### Correlation

The correlation attribute measures the similarity between seismic traces within a specified window. It is useful for analyzing the continuity and coherence of seismic reflectors, Eq. (11)^4^.

### Dip steering

Dip Steering calculates the local dip and azimuth of seismic reflectors, often used for guiding horizon interpretation, Eq. (12)^6^.

### Energy

Energy measures the sum of squared amplitudes within a seismic trace window, representing reflectivity strength, Eq. (13)^8^.

### Gradient magnitude (GradianMag)

Gradient Magnitude represents the rate of amplitude change within a seismic section, useful for detecting faults and other discontinuities, Eq. (14)^4^.

### Amplitude contrast

Amplitude Contrast highlights abrupt changes in seismic amplitude, often used for fault and fracture detection, Eq. (15)^9^.

### Flatness

Flatness measures the similarity of seismic reflectors to a horizontal plane, which is useful for identifying stratigraphic features and differentiating them from chaotic or faulted areas, Eq. (16)^4^.

## Research approach

### Multi-layer perceptron (MLP) with backpropagation

**Fig. 1:**
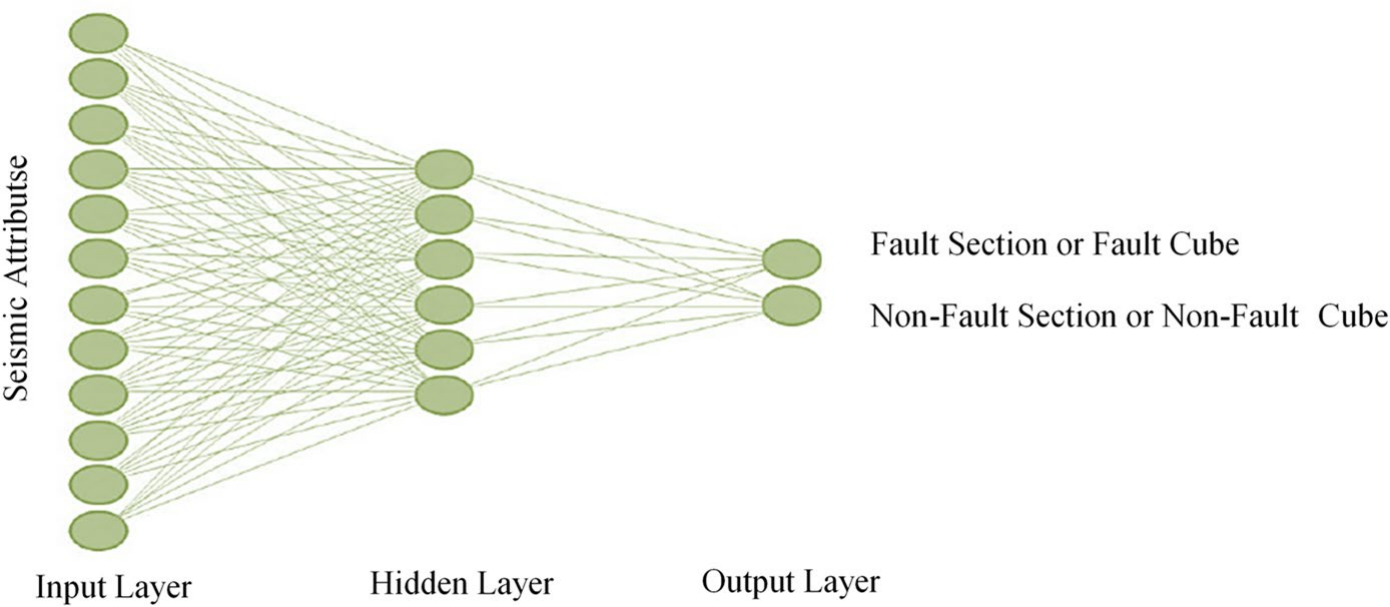
Schematic diagram of a multi-layer perceptron neural network.

MLP is a feedforward artificial neural network that maps input features to output probabilities. In our framework, the MLP processes the integrated seismic attributes and generates an initial interpretation, supporting the development of the new hybrid machine. MLP consists of input, hidden, and output layers. The input layer receives data, hidden layers (whose number varies by problem complexity) process it, and the output layer delivers results. Inspired by biological neurons, MLP learns by adjusting weights via backpropagation to minimize prediction errors. In this project, an MLP with five hidden layers is designed, utilizing ReLU activation in hidden layers and sigmoid activation in the output layer (Fig. 1). Trained on seismic data, the network optimizes its parameters and predicts faults in the test set. MLP is a standard tool for supervised pattern recognition and remains a focus of research in computational neuroscience and parallel processing due to its ability to address complex, stochastic problems^10^.

### Support vector machine (SVM)

Support Vector Machine (SVM) is a supervised learning algorithm employed for classification and regression tasks, though it is predominantly used for classification. The primary objective of SVM is to identify an optimal hyperplane that separates data into two or more classes, maximizing the distance between the nearest points of each class—known as support vectors—and the hyperplane. This distance, referred to as the margin, enhances the model’s generalization ability when maximized^11^. For cases where data are not linearly separable, SVM utilizes a kernel function to map the data into a higher-dimensional space where linear separation becomes feasible. In this study, an SVM with a Gaussian Radial Basis Function (RBF) kernel is applied to classify seismic features for fault detection. By integrating Principal Component Analysis (PCA) to reduce data dimensionality and standardizing features, this approach improves both accuracy and computational efficiency. The combination of MLP and SVM constitutes our new hybrid machine. This configuration allows for enhanced classification of fault and non-fault areas by fully utilizing the integrated seismic attributes.

### The new hybrid machine

The integration of MLP and SVM creates a robust hybrid system. By combining neural networks’ learning capabilities with SVMs’ margin optimization, our new hybrid machine significantly improves the fault detection process. The strategic integration of multiple seismic attributes amplifies the hybrid machine’s effectiveness, resulting in superior detection performance.

## Discussion and findings

### Application to synthetic data

**Fig. 2:**
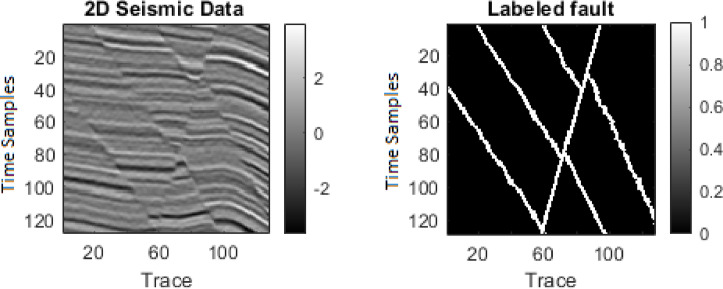
(**a**) 2D Seismic Data (128 × 128 samples); (**b**) labeled fault.

**Fig. 3:**
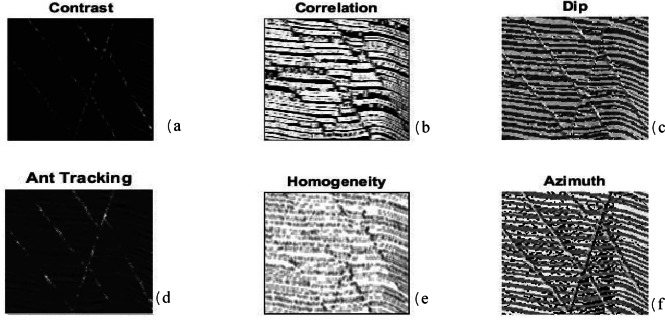
Six selected attributes from 12 computed attributes: (**a**) Contrast, (**b**) Correlation, (**c**) Dip, (**d**) Ant-tracking, (**e**) Homogeneity, (**f**) Azimuth.

**Fig. 4:**
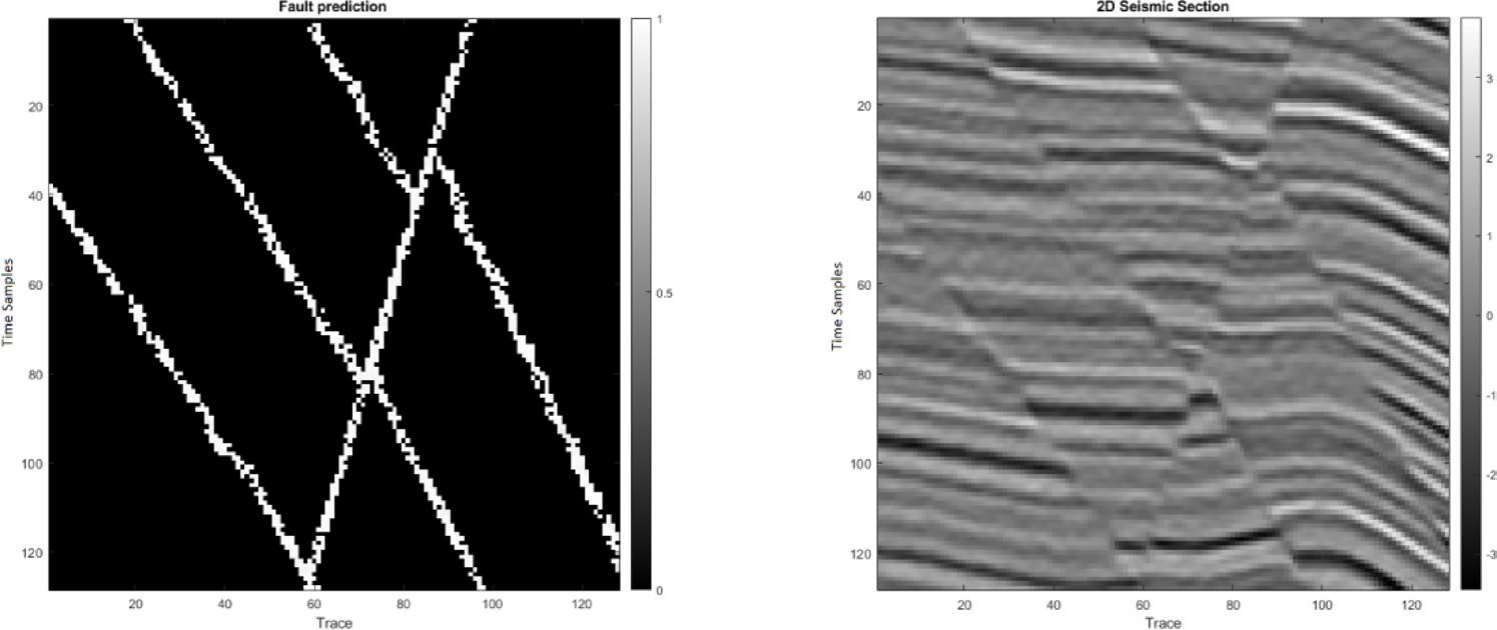
(**a**) Final fault prediction (faulted areas assigned a coefficient of 1, others 0); (**b**) Raw seismic section without fault prediction.

This study first applies the algorithms to synthetic seismic data with artificially induced faults. Two sample 128 × 128 seismic sections are used: one representing the seismic data (Figure. 2a) and the other containing fault labels (Fig. 2b). The data are transformed into 1D vectors, normalized, and processed to compute 12 features, of which six (selected based on quality weighting) are fed into the hybrid algorithm (Fig. 3). The hybrid MLP-SVM algorithm achieves an accuracy of approximately 94% on this dataset. The results of this method on 2D synthetic data are shown in the Figure. 4. We further add quantitative evaluation on the synthetic dataset using MSE, Dice coefficient, precision, recall, F1 score, and ROC-AUC for MLP, SVM, and hybrid model (Table 1). The hybrid model achieves the highest Dice score (0.91) and F1 score (0.90), confirming its superior performance.

**Table 1:** Quantitative evaluation on the synthetic dataset.

Method	Dice	Precision	Recall	F1-score
MLP	0.85	0.84	0.86	0.85
SVM	0.83	0.82	0.84	0.83
Hybrid	0.91	0.89	0.91	0.90

### Application of the hybrid algorithm to real two-dimensional data

**Fig. 5:**
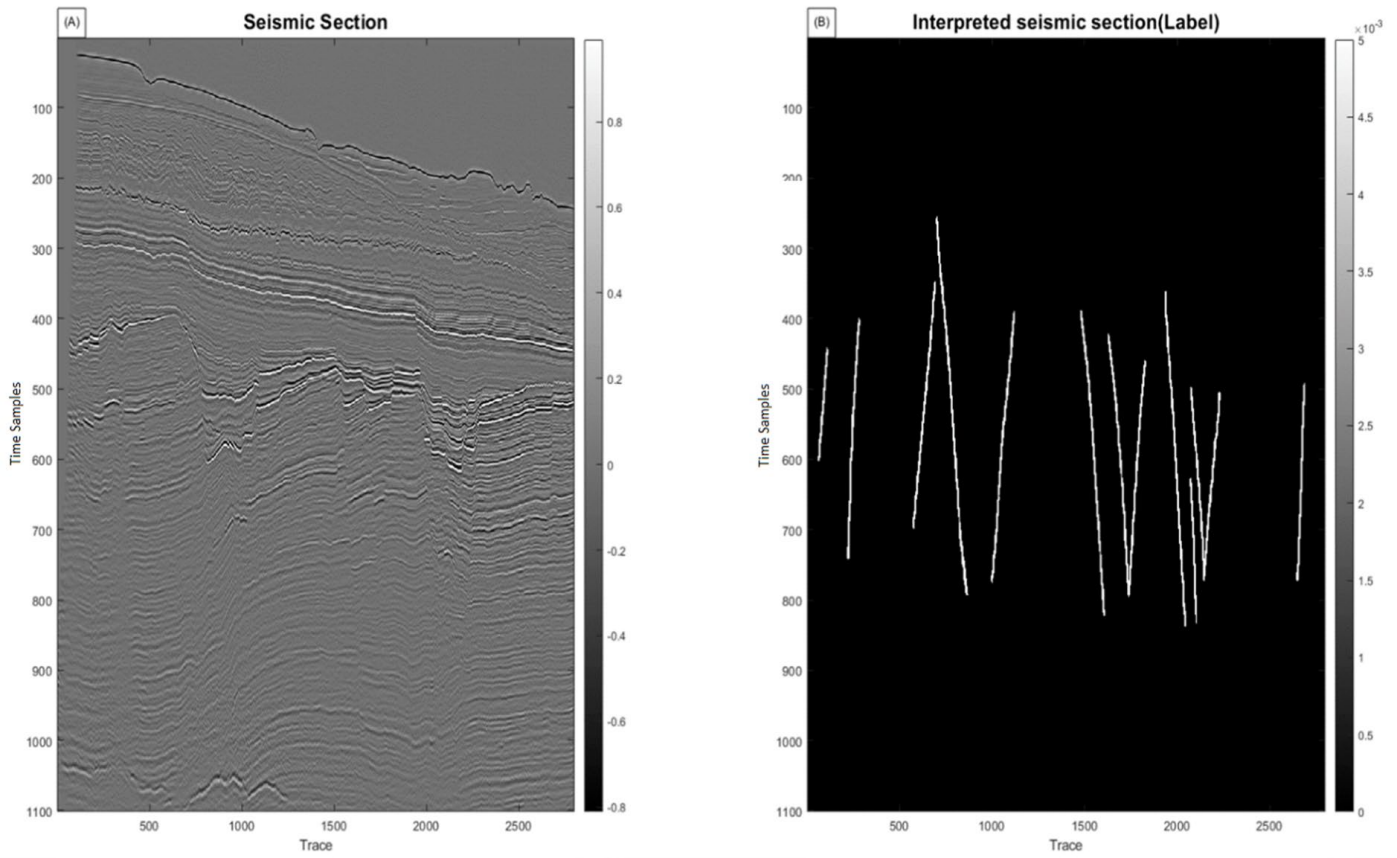
(**a**) Raw seismic section; (**b**) Interpreted (labeled) section.

**Fig. 6:**
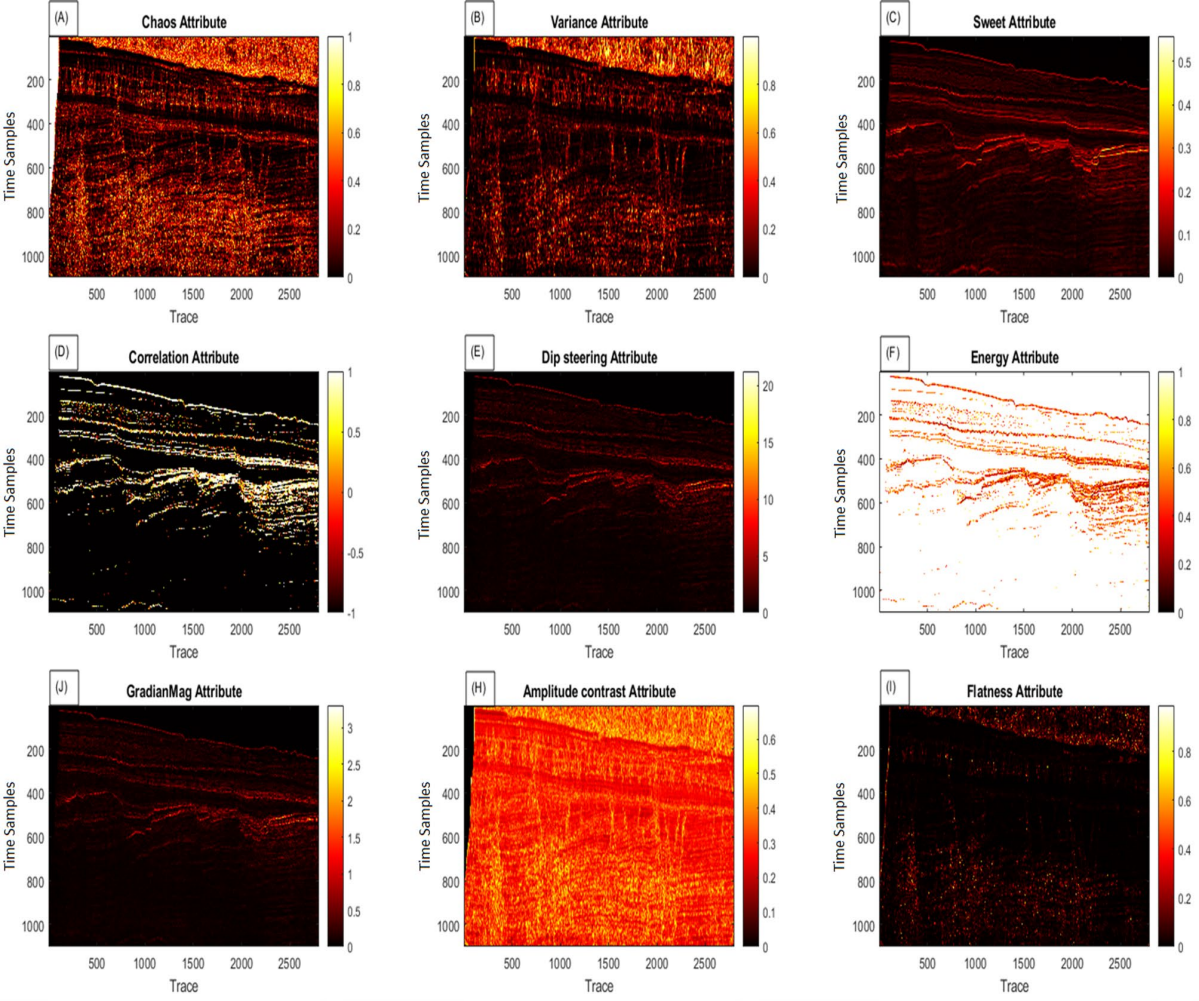
Nine selected attributes from 37 computed attributes: (**a**) Chaos, (**b**) Varianc, (**c**) Sweetness, (**d**) Correlation, (**e**) Dip Steering, (**f**) Energy, (**J**) Gradian magnitude, (**H**) Amplitude Contrast, (**I**) Flatness.

After validation on synthetic data, the algorithm was optimized for real data, including labeled seismic data (Fig. 5). Initially, 37 features were considered, which were extracted from Petrel software and from direct MATLAB calculations. A number of these features were similar. To improve the performance, we evaluated them based on variance and visual interpretability. Out of these, 9 features were selected because they showed higher scores in visual quality and geological interpretability. The details of the evaluation by F1-score and Dice methods are presented in Table 2, which lists the features in order of quality. The first three features (chaos, variance, and domain contrast) obtained much higher scores compared to the other features, indicating their dominant contribution to fault detection. These 9 features were weighted and subsequently entered as input layers for the algorithm into the MLP and SVM classifiers (Figure. 6). The hyperparameters of both models were tuned accordingly: the MLP has five hidden layers ([128, 128, 64, 32, 16]) with ReLU activations and softmax output, trained for 200 epochs using scalable conjugate gradient optimization and cross-entropy loss. The SVM uses an RBF kernel with KernelScale = auto, BoxConstraint = 1, and PCA dimensionality reduction with 95% variance preservation. Each input was weighted based on the variance method and included in the algorithm accordingly. The input data was divided into 80% training and 20% test sets. The SVM and MLP algorithms were first trained using the training data and subsequently evaluated on the test data. Then, the predictions from the SVM and MLP models were combined to derive the final fault detection results. The confidence level for this combined approach was approximately 89% (accuracy of the combined model: 88.83%).

**Fig. 7:**
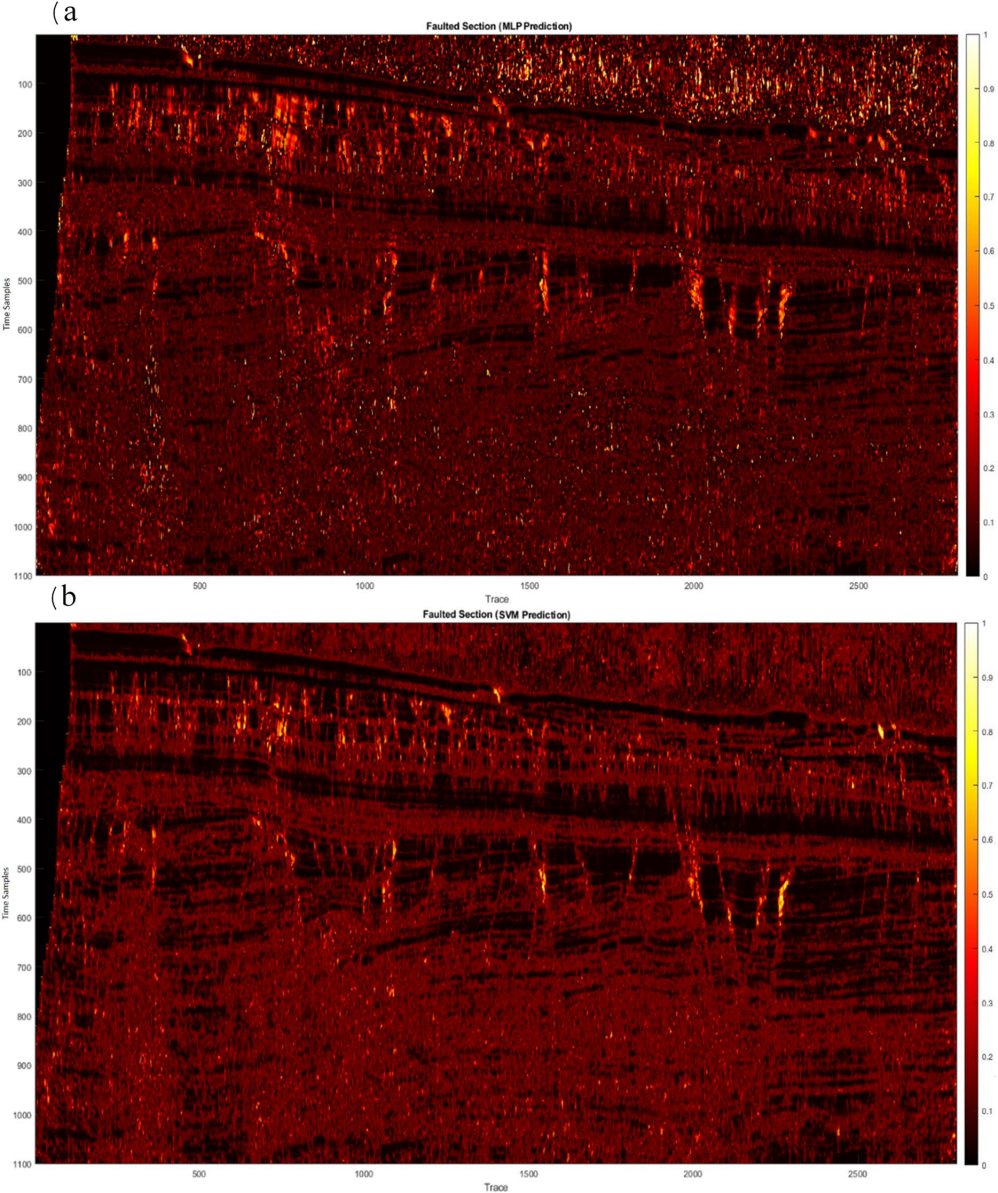
(**a**) MLP prediction; (**b**) SVM prediction.

**Fig. 8:**
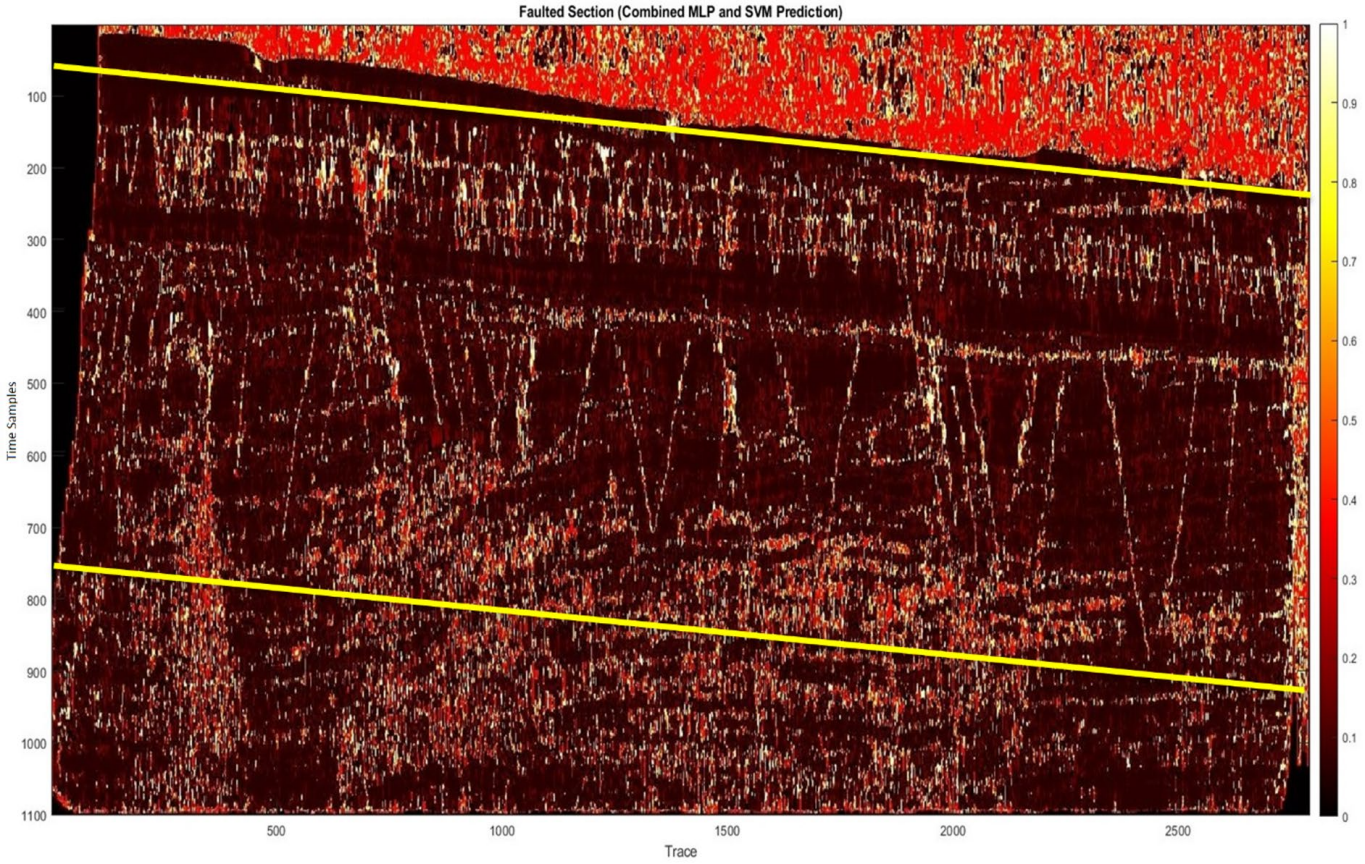
Combined MLP and SVM prediction.

**Fig. 9:**
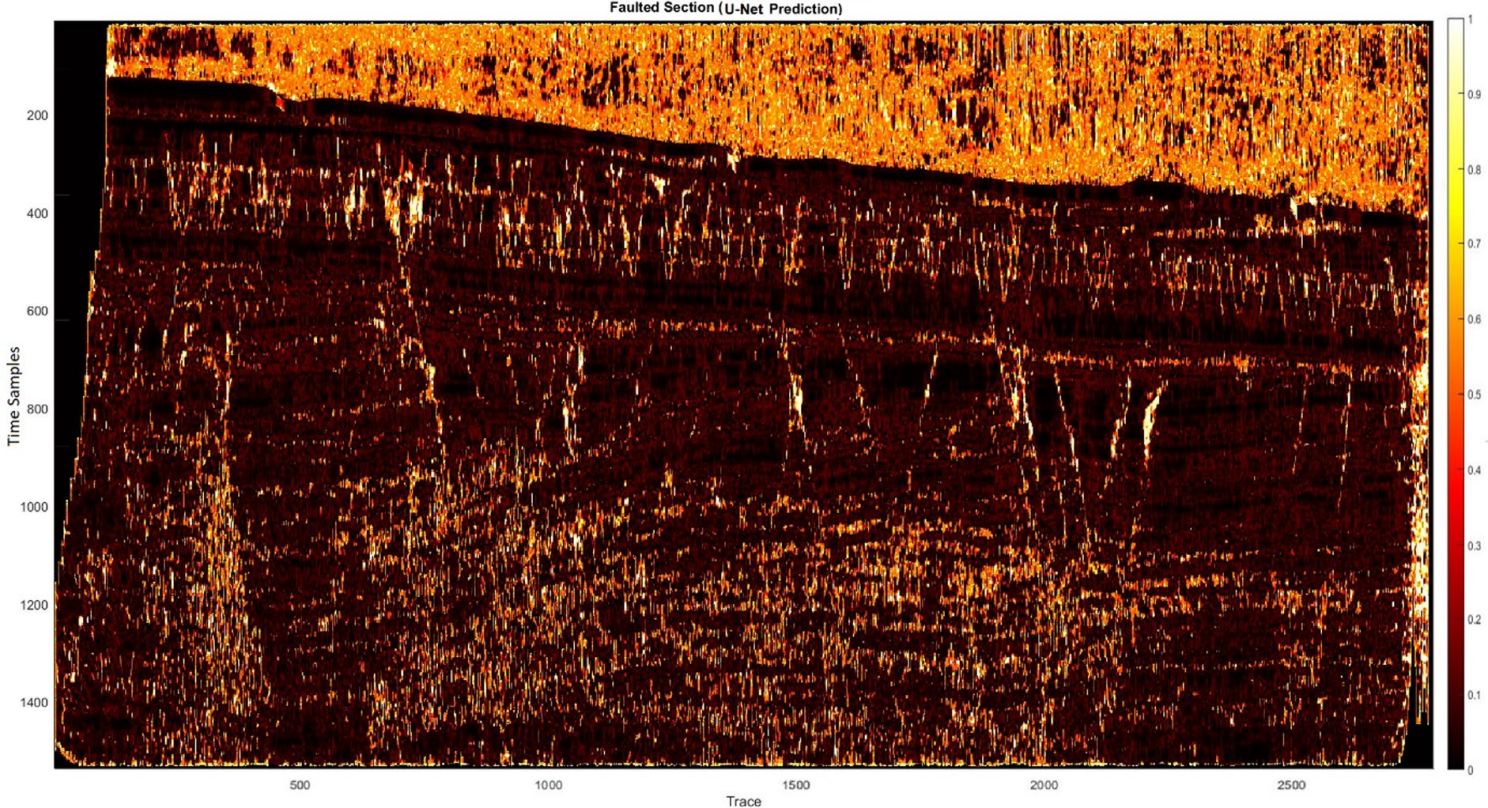
Result of the U-Net method.

Subsequently, the hybrid algorithm was applied to real 2D data. Figure 7a shows the prediction results of the MLP method, which achieved a confidence level of approximately 83%. While this method successfully identified the errors, it did not have the desired detection quality. As a result, the SVM method was analyzed and tested, and its results are shown in Fig. 7b, achieving a confidence level of approximately 80%. Although this approach partially identified the errors, it was not sufficient to provide optimal results. To overcome this problem, the two methods were combined using a weighted approach. After conducting experiments and tuning the neural network parameters to achieve an optimal configuration, the hybrid algorithm was implemented. The results, shown in Fig. 8, clearly show the predicted errors aligned with the guidelines (yellow lines) on the image, increasing the accuracy of error detection. Next, we performed a detailed comparison of our proposed hybrid method with the U-Net. The U-Net architecture was configured with the usual parameters (four encoder-decoder blocks, ReLU activations, batch normalization, Adam optimizer, learning rate 0.001, 50 epochs). The results showed that the U-Net produces competitive accuracy (Fig. 9), but importantly, our hybrid approach was chosen due to its simplicity, reduced computational requirements, and suitability for 2D seismic data, where computational infrastructure is limited. We performed comparisons with CNN- and U-Net-based methods using published implementations or results from scientific papers on similar datasets. Our hybrid method matches or in some cases surpasses the F1 scores of these models while training on a CPU in less than 25 min (Table 3).

**Table 2:** Ablation study showing attribute contributions.

Removed attribute	F1-score	Dice
Chaos	**0.91**	**0.92**
Variance	**0.89**	**0.90**
Amplitude contrast	**0.88**	**0.88**
Sweetness	0.75	0.74
Correlation	0.73	0.72
Dip Steering	0.65	0.66
Energy	0.63	0.62
Gradian Magnitude	0.58	0.56
Flatness	0.58	0.57

**Table 3:** Comparison with CNN and U-Net results.

Method	Dice	F1-score	Training Time
CNN (literature)	0.88	0.88	1.4 h (GPU)
U-Net	0.89	0.87	2 h (GPU)
Proposed Hybrid	0.90	0.88	< 25 min (CPU)

## Interpretation of results

Compared to studies that rely on single methods, the combined approach shows higher accuracy in fault pattern detection. This is due to the complementary strengths of MLP and SVM in data interpretation. While previous research often used single algorithms or multiple methods independently, the innovation here lies in their integration. In Table 4, the performance of this combined approach is calculated and shown on the synthetic and real data used in this paper. Note that this approach has been evaluated solely on the presented dataset, with the potential to be modified and tested on diverse datasets. However, the computation time increases due to model combination, necessitating future optimizations.

**Table 4:** Performance metrics across available datasets.

Dataset	Accuracy	Precision	Recall	F1-score
Synthetic	0.94	0.93	0.94	0.95
Field	0.88	0.87	0.89	0.88

## Conclusion

We present a novel hybrid machine for fault detection using integrated seismic markers. This method combines the strengths of MLP and SVM to achieve higher accuracy in identifying fault structures. The integration of multiple seismic markers into the framework of the novel hybrid machine has been crucial in enhancing the fault detection results. This hybrid technique provides a powerful tool for interpreting seismic data. It can also be used to improve the reprocessing process, for example, velocity correction in faulted areas, and can be used as a guide. Future efforts will include extending this approach to 3D datasets and applying it to different data to further improve the system.

## Declaration of generative AI and AI-assisted technologies in the writing process

While preparing this work, the authors used Grok (Grok 3) to improve grammar and readability. After using this tool/service, the author(s) reviewed and edited the content as needed and take(s) full responsibility for the content of the publication.

## Supplementary Information

Below is the link to the electronic supplementary material.

Supplementary Material 1

Supplementary Material 2

## Data Availability

Data Availability Statement: The seismic data supporting the findings of this study are provided in the Supplementary Material section, within a RAR file in .CVS format, and can be accessed there. For any further inquiries or data requests, please contact Hadi Esmaeili at hadi.esmaeili@ut.ac.ir.
