# Supplementary material for: Integrating multiple seismic attributes for fault detection using a new hybrid machine learning
Source: Sci Rep. 2025 Nov 28;15:42744. doi: 10.1038/s41598-025-26889-y (PMC12663330; doi:10.1038/s41598-025-26889-y)
Supplement: Supplementary file 1 — Supplementary Material 1 [file 41598_2025_26889_MOESM1_ESM.docx]

**Appendix**

**Curvature Attribute**

$$z=ax^{2}+by^{2}+cxy+dx+ey+f (1)$$

Here, z represents the depth or reflection time at the point of interest, while x and y denote horizontal coordinates. Coefficients a,b,c,d,e, and f are derived by fitting the surface to the central point and its neighbors, providing geometric insights into surface curvature. Various curvature types (e.g., mean, maximum, minimum, Gaussian) are then calculated using these coefficients [Roberts, 2001].

##### **Gray-Level Co-occurrence Matrix (GLCM)**

$$\text{Contrast }=\sum_{i,j} (i-j)^{2}\cdot P\left( i,j \right) (2)$$

$$\text{Correlation }=\sum_{i,j} {\left( i-\mu_{i} \right)\left( j-\mu_{j} \right)\cdot P(i,j)}/{\sigma_{i}\sigma_{j}} (3)$$

$$\text{Energy }=\sum_{i,j} P(i,j)^{2} (4)$$

$$\text{Homogeneity }=\sum_{i,j} {P(i,j)}/{1+|i-j|} (5)$$

where p(i,j) is the probability of co-occurrence of gray levels i and j, μ is the mean, and σ is the standard deviation of gray-level intensities [Haralick et al.,1973].

**Dip and Azimuth Attributes**

$$Dip=\arctan\left( \sqrt{\left( {\partial z}/{\partial x} \right)^{2}+\left( {\partial z}/{\partial y} \right)^{2}} \right) (6)$$

$$\text{Azimuth }=\arctan\left( ({\partial z}/{\partial y)}/({\partial z}/{\partial x)} \right) (7)$$

where ${\partial z}/{\partial y}$ and ${\partial z}/{\partial x}$​ are gradients in the x and y directions, respectively.

### Chaos

$$\text{Chaos }=1-( {\lambda_{1}}/{\sum_{i=1}^{3} \lambda_{i}}) (8)$$

Where λ1, λ2 and λ3(lambda_1, lambda_2, lambda_3) are the eigenvalues from the structure tensor [Chopra & Marfurt, 2007].

### Variance

$$\text{Variance}=1\text{ }-\left( \text{Mean of Amplitude Values}/\text{Local Amplitude Values} \right)^{2} (9)$$

### Sweetness (Sweet)

$$\text{Sweetness }={A(t)}/\sqrt{F(t)} (10)$$

Where A(t) is the Instantaneous amplitude and F(t) is the Instantaneous frequency [Radovich & Oliveros, 1998].

### Correlation

$$\text{Correlation }= {\sum\left( x_{i}-\bar{x} \right)\left( y_{i}-\bar{y} \right)}/{\sqrt{\sum\left( x_{i}-\bar{x} \right)^{2}\sum\left( y_{i}-\bar{y} \right)^{2}}} (11)$$

Where $x_{i}$, $y_{i}$, are seismic trace amplitudes and $\bar{x}$, $\bar{y}$ is Mean amplitudes [Chopra & Marfurt, 2007].

### Dip Steering

$$\theta=\tan^{-1} \left( {\partial T}/{\partial x},{\partial T}/{\partial y} \right) (12)$$

Where T is Two-way travel time (TWT) and x and y are Spatial coordinates [Marfurt et al., 1998].

### Energy

Energy measures the sum of squared amplitudes within a seismic trace window, representing reflectivity strength.

$$\text{Energy }=\sum A_{i}^{2} (13)$$

Where Ai is the Amplitude of the seismic sample [Taner & Sheriff, 1977].

### Gradient Magnitude (GradianMag)

$$\left| \nabla A \right|=\sqrt{\left( {\partial A}/{\partial x} \right)^{2}+\left( {\partial A}/{\partial y} \right)^{2}} (14)$$

Where A is the Amplitude of seismic data [Chopra & Marfurt, 2007].

### Amplitude Contrast

$$\text{Contrast }=\left| A_{i}-A_{j} \right|/{A_{i}+A_{j}} (15)$$

Where Ai and Aj are Amplitude values from neighboring seismic samples [Marfurt, 2006].

### Flatness

$$\text{Flatness }=1-( {\sigma_{\text{dip }}}/{\sigma_{\text{horiz }})} (16)$$

Where $\sigma_{\text{dip }}$ is the standard deviation of dip values and $\sigma_{\text{horiz }}$ is the standard deviation of horizontal reflectors [Chopra & Marfurt, 2007].
